# Increased whole body energy expenditure and protection against diet-induced obesity in Cyp8b1-deficient mice is accompanied by altered adipose tissue features

**DOI:** 10.1080/21623945.2020.1827519

**Published:** 2020-10-04

**Authors:** Ulrika Axling, Michele Cavalera, Eva Degerman, Mats Gåfvels, Gösta Eggertsen, Cecilia Holm

**Affiliations:** a Lund University Diabetes Centre, Department of Experimental Medical Science, Lund University, Lund, Sweden; b Department of Laboratory Medicine, Lund University, Lund, Sweden; c Department of Laboratory Medicine, Karolinska Institute, Karolinska University Hospital Huddinge, Stockholm, Sweden

**Keywords:** Bile acids, lipolysis, lipogenesis, insulin sensitivity, glucose tolerance, energy expenditure, white adipocytes, brown adipocytes, insulin secretion

## Abstract

The aim of this study was to elucidate mechanisms whereby bile acids exert beneficial metabolic effects, using the *Cyp8b1^−/-^* mouse as model. These mice are unable to synthesize cholic acid, resulting in increased synthesis of chenodeoxycholic acid and enlarged bile acid pool. *Cyp8b1^−/-^* mice were found to be protected against high-fat diet induced obesity. Bomb calorimetry measurements showed increased faecal energy output in *Cyp8b1^−/^*mice. Indirect calorimetry measurements demonstrated increased energy expenditure in *Cyp8b1^−/-^* mice. Meal tolerance tests revealed no differences in glucose disposal, but the insulin response was lower in *Cyp8b1^−/-^* mice. Intravenous glucose tolerance tests, as well as static incubations of isolated islets, showed no difference between the groups, whereas insulin tolerance tests demonstrated improved insulin sensitivity in *Cyp8b1^−/-^* mice. The genes encoding mitochondrial transcription factor A (TFAM) and type 2-iodothyronine deiodinase were upregulated in brown adipose tissue of *Cyp8b1^/-^* mice and Western blot analyses showed increased abundance of TFAM, and a trend towards increased abundance of UCP1. The upregulation of TFAM and UCP1 was accompanied by increased mitochondrial density, as shown by transmission electron microscopy. White adipocytes of *Cyp8b1^−/-^* mice exhibited increased responsiveness to both catecholamines and insulin in lipolysis experiments and increased insulin-stimulated lipogenesis. In conclusion, increased energy expenditure, mitochondrial density of brown adipocytes and faecal energy output may all contribute to the protection against diet-induced obesity of *Cyp8b1^−/-^* mice. Enhanced insulin sensitivity of *Cyp8b1^−/-^* mice is accompanied by increased hormonal responsiveness of white adipocytes.

## Introduction

In addition to their well-known role in intestinal lipid absorption, it has become increasingly clear that bile acids also exert metabolic effects through their action as signalling molecules in a variety of tissues (for a recent review see [1]). The two major receptors for bile acids are the farnesoid X receptor (FXR) and the G-protein-coupled bile acid receptor 1 (TGR5), both of which are expressed in a tissue-dependent manner [2]. Each bile acid binds to the respective receptor with different affinity depending on its physicochemical properties. Thus, the size and the composition of the plasma bile acid pool, together with the expression levels of FXR and TGR5 in target cells, are important determinants for the metabolic effects of bile acids. In addition, intestinal bile acids can exert metabolic actions via FXR-dependent effects on the expression and secretion of FGF19 (FGF15 in mice), which is a hormone binding to FGF receptors in target cells. Mouse models represent valuable tools in ongoing research aimed to describe the metabolic effects of bile acids and the mechanisms underlying these. One such model is the *Cyp8b1^−/-^* mouse, which lacks steroid 12-alpha hydroxylase and therefore is unable to synthesize cholic acid [3]. This results in increased synthesis of chenodeoxycholic acid (CDCA), enlargement of the bile acid pool in the gallbladder [3], as well as in plasma [4], and reduced intestinal absorption of cholesterol. Moreover, muricholic acids are accumulated in *Cyp8b1^−/-^* mice, since mice, as opposed to humans, are able to synthesize 6-hydroxylated muricholic acid from CDCA. Several recent studies have shown that *Cyp8b1^−/-^* mice are protected against high-fat diet induced weight gain, glucose intolerance and liver steatosis [3–7]. The aim of the present study was to elucidate mechanisms whereby loss of Cyp8b1, and thereby cholic acid, exert these beneficial metabolic effects.

## Materials and methods

### Animals

The *Cyp8b1^−/-^* mouse [3] was embryo transferred to the animal facility at Lund University from the Karolinska Institute and backcrossed to a C57Bl/6 background for nine generations. Initially, we attempted to set up the breeding using homozygous animals only. However, this resulted in high lethality of the pups after birth and we therefore had to switch to heterozygous mating. The pups were separated according to genotype as soon as possible after weaning. Unless otherwise stated, male *Cyp8b1^−/-^* mice and their *Cyp8b1^+/+^* littermates from heterozygous mating, were used. The mice were fed a 60 kcal% high-fat diet (HFD, D12492, Research diets Inc.) for 10–14 weeks. The mice were 9–14 weeks of age at the start of the HFD-feeding and were housed in a temperature-controlled room (21°C) with a 12-h light to dark cycle with free access to food and water. Body weight was monitored weekly. Body composition was determined with dual-energy X-ray absorptiometry (DEXA) using a Lunar PIXImus (Madison, WI, USA) [8]. The study was approved by the Regional Ethical Committee of Malmö/Lund, Sweden.

### Faecal energy content

The gross energy content in faeces was determined with a bomb calorimeter (1108P Oxygen Bomb, Parr Instrument Company, Moline, IL, USA) following the manufacturer’s instruction. Faeces were collected from single-caged mice over 24 h and freeze-dried for 24 h prior to analysis.

### Indirect gas calorimetry and behavioural monitoring

Whole-body energy metabolism, food, and water intake as well as voluntary locomotor and wheel activity were measured with PhenoMaster/LabMaster Home cage System (TSE-systems, Bad Homburg, Germany). Mice were acclimatized for 2 days prior to actual measurements. All parameters were recorded every 15 min for 24 h at 22°C. Two 24-h experiments were carried out and averaged for each mouse.

### Mixed meal tolerance test

A mixed meal tolerance test was performed in 4-h fasted mice (8 a.m. −12 noon). A mixture of glucose (60 E%, Sigma), whey protein (20 E%, SELF Omninutrition), and peanut oil (20 E%, Zeta), with a total caloric content of 0.285 kcal, was administered via gavage. Blood samples were taken from the saphenous vein at 0, 15, 30, 60, and 90 min. Blood was collected in heparin-coated tubes and centrifuged for 10 min at 1000 x g, plasma was collected and frozen at −20°C until analysis of insulin using an ELISA method (Mercodia, 10–1247-10). Blood glucose was measured at the indicated time points with Accu-check Aviva (Roche Diagnostic Gmbh, Mannheim, Germany).

### Intravenous glucose tolerance test

An intravenous glucose tolerance test (IVGTT) was performed in 6-h fasted (7 a.m.-1 p.m.) anesthetized mice by injection of 0.75 mg/kg D-glucose in the tail vain. Mice were anesthetized with 0.5 mg of Fluanison, 0.02 mg of fentanyl (Hypnorm, Janssen, Vm 41760/4000) and 0.25 mg of midazolam (Dormicum, Hoffman-LaRoche, Vnr 411389) per mouse. Blood samples were taken by orbital puncture at 0, 1, 5, 10, 20, 30 and 50 min after the glucose injection. Blood glucose was measured at the indicated time points with Accu-check Aviva (Roche Diagnostic Gmbh, Mannheim, Germany). After immediate centrifugation, plasma was collected and kept frozen at −20°C until analysis of insulin using an ELISA method (Mercodia, 10–1247-10).

### Islet isolation and insulin secretion

Insulin secretion from freshly isolated islets was measured as described [9]. Islets were isolated by standard collagenase digestion and handpicked under a stereo microscope. The islets were first allowed to recover in HEPES balanced salt solution (HBSS, 114 mM NaCl, 4.7 mM KCl, 1.2 mM KH_2_PO_4_, 1.16 mM MgSO_4_, 20 Mm HEPES, 2.5 mM CaCl_2_, 25.5 mM NaHCO_3_, 0.1% bovine serum albumin, pH 7.2) containing 5.6 mM glucose for 60 min in an incubator at 37°C and 5% CO_2_. Batches of three islets were transferred to a 96-well plate containing 200 µl of HBSS per well with an addition of 2.8, 11.1 (± 100 nM GLP-1) or 16.7 mM glucose. After another 60 min of incubation, a sample was removed for measurement of insulin by an ELISA method (Mercodia, 10–1247-10).

### Insulin tolerance test

An intraperitoneal insulin tolerance test (IPITT) was performed on 4-h fasted mice (8 a.m. −12 noon) by injecting 0.75 U/kg of insulin (Actrapid**®**, Novo Nordisk). Blood samples were collected at 0, 15, 30 and 60 min and blood glucose levels were determined with Accu-check Aviva (Roche Diagnostic Gmbh, Mannheim, Germany).

### RNA preparation and real-time quantitative PCR

Total RNA was isolated from the tissue using Qiazol lysis reagent (Qiagen Sciences, 79306). Two microgram of RNA were treated with DNase I (DNase I amplification grade, Invitrogen, 18068015) and then reversely transcribed using random hexamers (Invitrogen, N8080127) and SuperScript II reverse transcriptase (Invitrogen, 18064014). Quantitative real-time polymerase chain reactions were performed using ABI PRISM 7900 System using TaqMan Fast Advanced MasterMix (4444557, Applied Biosystems) and the following primers: Bmp7 (Mm00432102_m1), Ppargc1a (Mm01208835_m1), Fgf21 (Mm00840165_g1), Rps29 (Mm02342448_gH), Tbp (Mm01277042_m1), Tfam (Mm00447485_m1), Cited1 (Mm01235642_g1), FXR (Mm00436425_m1), TGR5 (Mm04212121_s1), D2 (Mm01184611_m1), Cyp7a1 (Mm00484150_m1), Cyp27a1 (Mm00470430_m1), Cyp8b1 (Mm00501637_s1), Nrf1 (Mm01135606_m1), Rps29 (Mm02342448_gH), Tbp (Mm01277042_m1). Qiagen SYBR green primers were Prdm16 (QT00148127), Ucp1 (QT00097300), Cpt1 (QT00172564), Tbp (QT00198443) and reagents (A25742, Applied Biosystems). Each reaction was performed in duplicate and results were normalized by geometric average of two internal controls (TBP and RPS29).

### Western blot analysis

Brown adipose tissue was homogenized in a buffer containing 50 mM TES (N166 [Tris(hydroxymethyl)methyl]-2-aminoethanesulfonic acid), pH 7.4, 250 mM sucrose, 1 mM EDTA, 2 mM ethylene glycol tetraacetic acid (EGTA), 40 mM phenylphosphate, 5 mM sodium fluoride, 1 mM dithiothreitol (DTT), 50 µM sodium vanadate, complete protease inhibitor cocktail (Roche) and 1% NP40 using a glass homogenizer (10 strokes; 3 ml buffer/gram of tissue). The lysates were centrifuged for 5 min at 5000 × g, 4°C and the supernatants were used for further analysis. Tissue lysates (10 µg protein as measured by Bradford) were mixed with LDS (Lauryl Dodecyl Sulphate) sample buffer 4X (Invitrogen) containing 300 mM DTT and subjected to electrophoresis on 4–12% bisacrylamide gels (Novex, Invitrogen). Proteins were transferred to Immobilon-P, PVDF (Polyvinylidene Fluoride) membranes (Merck Millipore), blocked with 10% milk in Tris-buffered saline tween-20 (50 mM Tris, pH 7.6, 150 mM NaCl and 0.1% Tween-20) for 30 min and incubated at 4°C overnight with primary antibodies against TFAM and UCP1 (ab 131607 and ab 10983, respectively, from Abcam). Membranes were incubated with secondary antibody conjugated with horseradish peroxidase (HRP) for 1 h at room temperature, incubated with SuperSignal West Pico ECL (enzymatic chemiluminescence) reagent (Thermo Scientific, Rockford, USA) for 3 min followed by imaging (Molecular Imager ChemiDoc XRS+, Bio-Rad Laboratories, Solna, Sweden) and quantification (Image lab software, V.3.0, Bio-Rad Laboratories). Ponceau S (Sigma-Aldrich) was used to stain membranes for total protein.

### Transmission electron microscopy

Interscapular brown adipose tissue (BAT) was excised from *Cyp8b1^−/-^* and *Cyp8b1^+/+^* mice, cut into 1.5 mm pieces and processed according to the following scheme. Following fixation in Sørensen’s phosphate buffer containing 1.5% glutaraldehyde and 1.5% paraformaldehyde over night at 4°C, specimens were post-fixed with osmium tetroxide (1% in PBS) for 2 h at room temperature, followed by washing three times with MilliQ-water for 1 h. Specimens were dehydrated using increasing concentrations of acetone, and embedded in Polybed 812 epoxy resin. Sections (1 µm) were cut and studied in a light microscope (CETI) to locate well-fixed tissue in blocks, for further trimming with a glass knife. Ultrathin sections (50 nm) were cut with a diamond knife using a Leica EM UC7 ultratome (Leica Microsystems,Wetzlar, Germany) and mounted on pioloform-filled copper slot grids. Finally, the grids were contrasted with 4% uranyl acetate solution and viewed under a 120 kV Tecnai Biotwin microscope (FEI, Hillsboro, OR, USA). For each specimen, 10 different, randomly chosen, areas were examined and photographed. The specimens were blinded to four investigators who independently analysed the images and divided the animals into two groups, based on mitochondrial morphology. Image J was used to analyse mitochondrial area of each image.

### Lipolysis

Isolated white adipocytes were obtained by digestion of epididymal adipose tissue in a collagenase solution (0.6 mg/ml) of Krebs-Ringer buffer (120 mM NaCl, 4.7 mM KCl, 1.2 mM KH_2_PO_4_, 2.5 mM CaCl_2_ and 1.2 mM MgSO_4_), supplemented with 25 mM HEPES, pH 7.4, 2 mM glucose and 3.5% BSA, at 37°C with shaking at 120 rpm for 60 min followed by shaking at 140 rpm for another 20 min. Cells were diluted to a concentration of 5% in Krebs-Ringer buffer containing 25 mM HEPES, 1% fatty acid-free BSA (Roche, 10775835001), 200 nM adenosine and 2 mM glucose. Isoproterenol and/or insulin was added to modulate lipolysis and cells were incubated at 37°C with shaking (150 rpm) for 30 min. Experiments were ended by incubating the cells on ice for 30 min before aliquots (150 µl) of the incubation media was taken for analysis of glycerol release. Glycerol concentration was determined using a commercially available kit with the addition of Amplex Ultra Red (Invitrogen, A36006), a hydrogen peroxide sensitive fluorescence dye, as described by Clark *et al*. [10].

### Lipogenesis

Isolated adipocytes were obtained by collagenase digestion as described above. Lipogenesis was determined as described [11] with some modifications. Aliquots (400 µl) of 2% (v/v) cell suspension in Krebs–Ringer buffer supplemented with 25 mM HEPES, pH 7.4, 0.55 mM glucose and 1% BSA were distributed into vials containing 0.4 µCi [6–^3^H]glucose (Amersham, TRK 85) and different concentrations of insulin (Actrapid**®** Novo Nordisk). Incubations were carried out for 45 min at 37°C while shaking (80 rpm). The reaction was stopped by addition of 3.5 ml toluol-based scintillation liquid containing 0.3 mg/ml POPOP (1,4-bis [5-Phenyl-2-oxazolyl] benzene; 2,2ʹ-p-Phenylenebis [5ʹ-phenyloxazole], Sigma, P3754) and 5 mg/ml PPO (2,5-Diphenyloxazole, Sigma, D210404). *De novo* lipid synthesis was determined by liquid scintillation counting of radioactivity incorporated into total cellular lipids.

### Statistics

Results are presented as means ± SD. Statistical analysis was performed with Graph Pad Prism 6 using nonparametric Mann-Whitney U test. Values of p < 0.05 were considered statistically significant.

## Results

### Cyp8b1^−/-^ *mice are protected against high-fat diet induced obesity and have increased faecal energy content*

**Figure 1.**
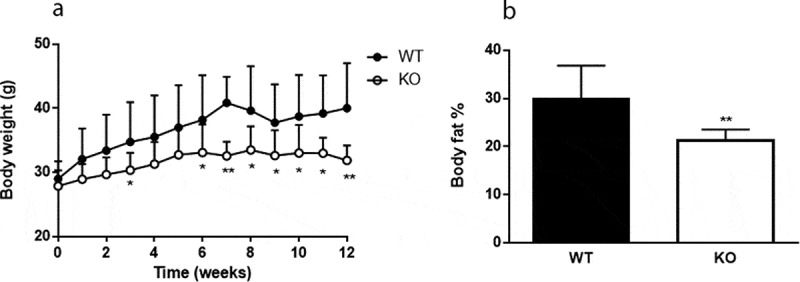
Cyp8b1^−/-^ (KO) mice are protected against diet-induced obesity. (a) Body weight curve during 12 weeks of high-fat diet (HFD) feeding of Cyp8b1^+/+^ (WT) and Cyp8b1^−/-^ (KO) mice. Data is shown as mean ± S.D, n = 16–17. (b) Body fat percent of Cyp8b1^+/+^ and Cyp8b1^−/-^ mice following 12 weeks of HFD feeding. Data is shown as mean ± SD, n = 7. ** = p < 0.01 and * = p < 0.05

*Cyp8b1^−/-^* mice gained significantly less body weight compared to *Cyp8b1^+/+^* mice on a HFD and this was accompanied by a reduction in body fat content (Figure 1). Lean body mass was not different between the two groups (data not shown). Food intake did not differ between *Cyp8b1^+/+^* and *Cyp8b1^−/-^* (2.6 ± 0.3 g/24 h vs. 2.4 ± 0.6 g/24 h). Faecal energy content was significantly increased in *Cyp8b1^−/-^* mice compared to *Cyp8b1^+/+^* (18.9 ± 0.38 vs. 17.5 ± 1.05 MJ/Kg, p < 0.01).

### Cyp8b1^−/-^ *mice have increased energy expenditure*

**Figure 2.**
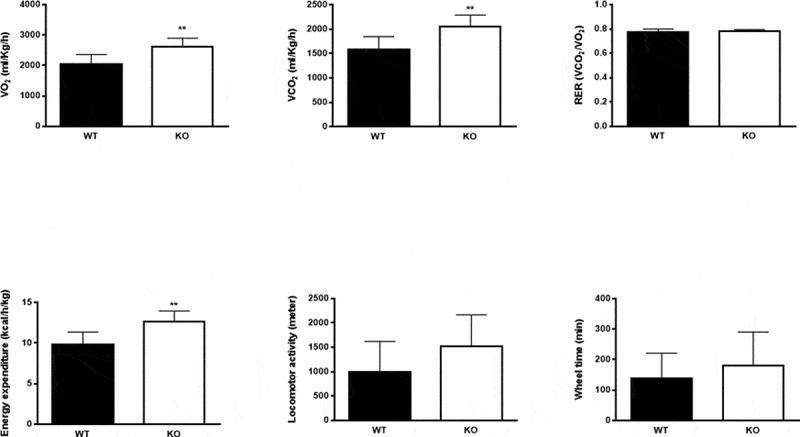
Energy expenditure is increased in Cyp8b1^−/-^ (KO) mice. Indirect calorimetry measurements of (a) volume of oxygen consumed (VO_2_), (b) carbon dioxide produced (VCO_2_), (c) respiratory exchange ratio (RER), (d) energy expenditure, (e) locomotor activity and (f) wheel time in HFD-fed Cyp8b1^+/+^ (WT) and Cyp8b1^−/-^ (KO) mice. Data is shown as mean ± SD, n = 8. ** = p < 0.01 and * = p < 0.05

Indirect calorimetry measurements showed a significant increase in oxygen consumption (VO_2_), carbon dioxide production (VCO_2_) and calculated energy expenditure in *Cyp8b1^−/-^* mice compared to *Cyp8b1^+/+^* (Figure 2). Respiratory exchange ratio (RER), however, did not differ between the two groups, indicating that metabolic fuel selection does not differ between *Cyp8b1^+/+^* and *Cyp8b1^−/-^* mice (Figure 2). Both locomotor activity and wheel running tended to be higher in *Cyp8b1^−/-^* compared to *Cyp8b1^+/+^* mice, but the difference did not attain statistical significance (Figure 2).

### Mixed meal tolerance and insulin sensitivity is enhanced in Cyp8b1^−/-^ mice

**Figure 3.**
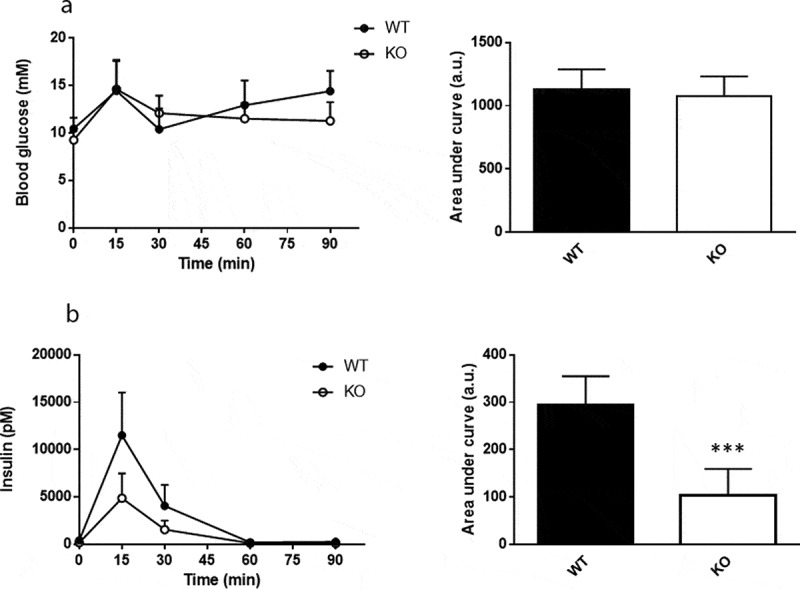
Enhanced meal tolerance in Cyp8b1^−/-^ (KO) mice. (a) Blood glucose levels and (b) plasma insulin levels in response to a meal tolerance test in HFD-fed 544 Cyp8b1^+/+^ (WT) and Cyp8b1^−/-^ (KO) mice. Data is shown as mean ± SD, n = 6–8. Area 545 under the curve is shown to the right of respective graph. *** = p < 0.001

**Figure 4.**
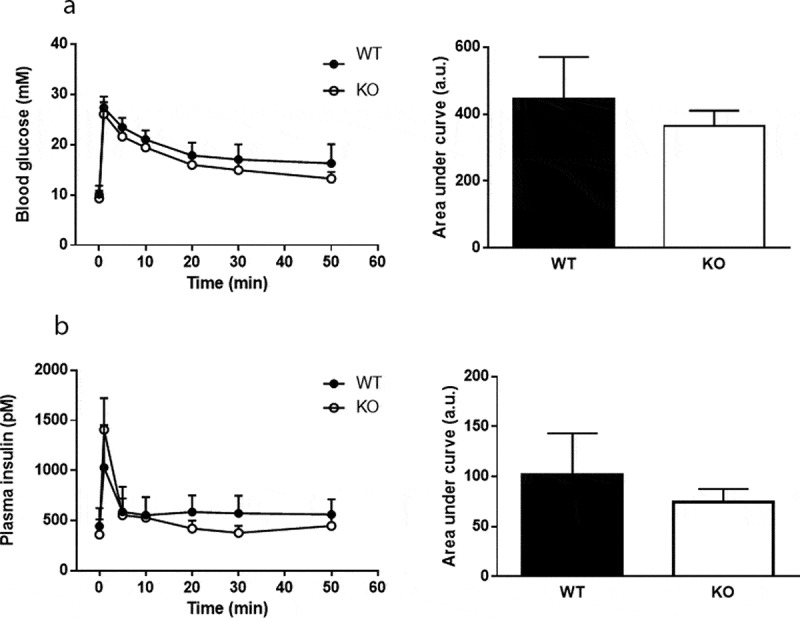
No difference in intravenous glucose tolerance between Cyp8b1^+/+^ (WT) and Cyp8b1^−/-^ (KO) mice. (a) Blood glucose levels and (b) plasma insulin levels in response to an intravenous glucose tolerance test in HFD-fed Cyp8b1^+/+^ (WT) and Cyp8b1^−/-^ (KO) mice. Data is shown as mean ± SD, n = 6–8. Area under the curve is shown to the right of respective graph

Plasma levels of glucose and insulin were measured in response to a mixed meal tolerance test. No difference in glucose disposal was found between *Cyp8b1^−/-^* and *Cyp8b1^+/+^* mice, but the insulin response was lower in *Cyp8b1^−/-^* (Figure 3). Next, an intravenous glucose tolerance test was performed. No differences in glucose disposal or insulin response to an intravenous glucose load were found between the two groups of mice (Figure 4). The basal glucose values were not different between the two groups (10.5 ± 1.4 vs. 10.1 ± 2.1 mmol/L).

**Figure 5.**
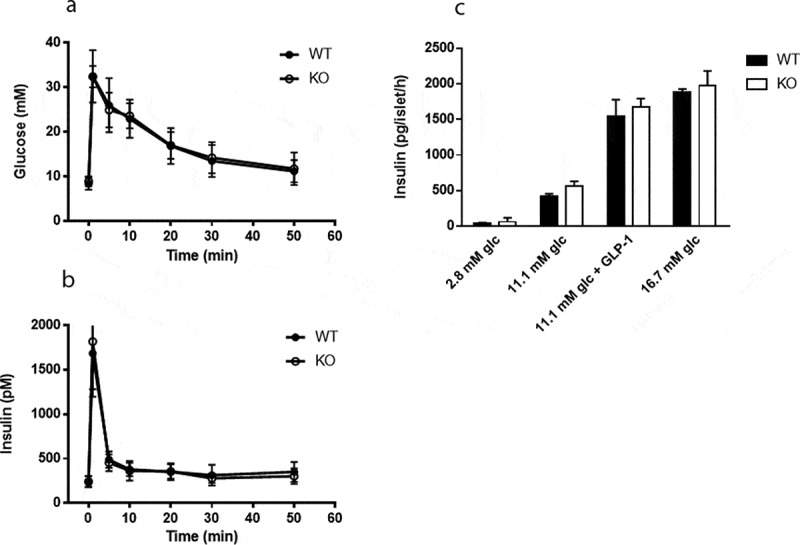
No difference in insulin secretion from isolated islets between Cyp8b1^+/+^ (WT) and Cyp8b1^−/-^ (KO) mice. (a) Blood glucose levels and (b) plasma insulin levels in response to an intravenous glucose tolerance test in HFD-fed male Cyp8b1^+/+^ (WT) and Cyp8b1^−/-^ (KO) mice from homozygous breeding. (c) Insulin 555 secretion from islets isolated from HFD-fed female Cyp8b1^+/+^ (WT) and Cyp8b1^−/^556 (KO) mice from homozygous breeding. Data is shown as mean ± SD, n = 10–13 (a,b) 557 and 3 (C), respectively

To assess islet function directly, static incubations of isolated islets were performed. There was no difference in insulin secretion between *Cyp8b1^+/+^* mice and *Cyp8b1^−/^*mice at any of the glucose concentrations analysed (Figure 5). Moreover, addition of the incretin GLP-1 to the intermediate glucose concentration exacerbated insulin secretion to the same extent in both strains of mice (Figure 5).

**Figure 6.**
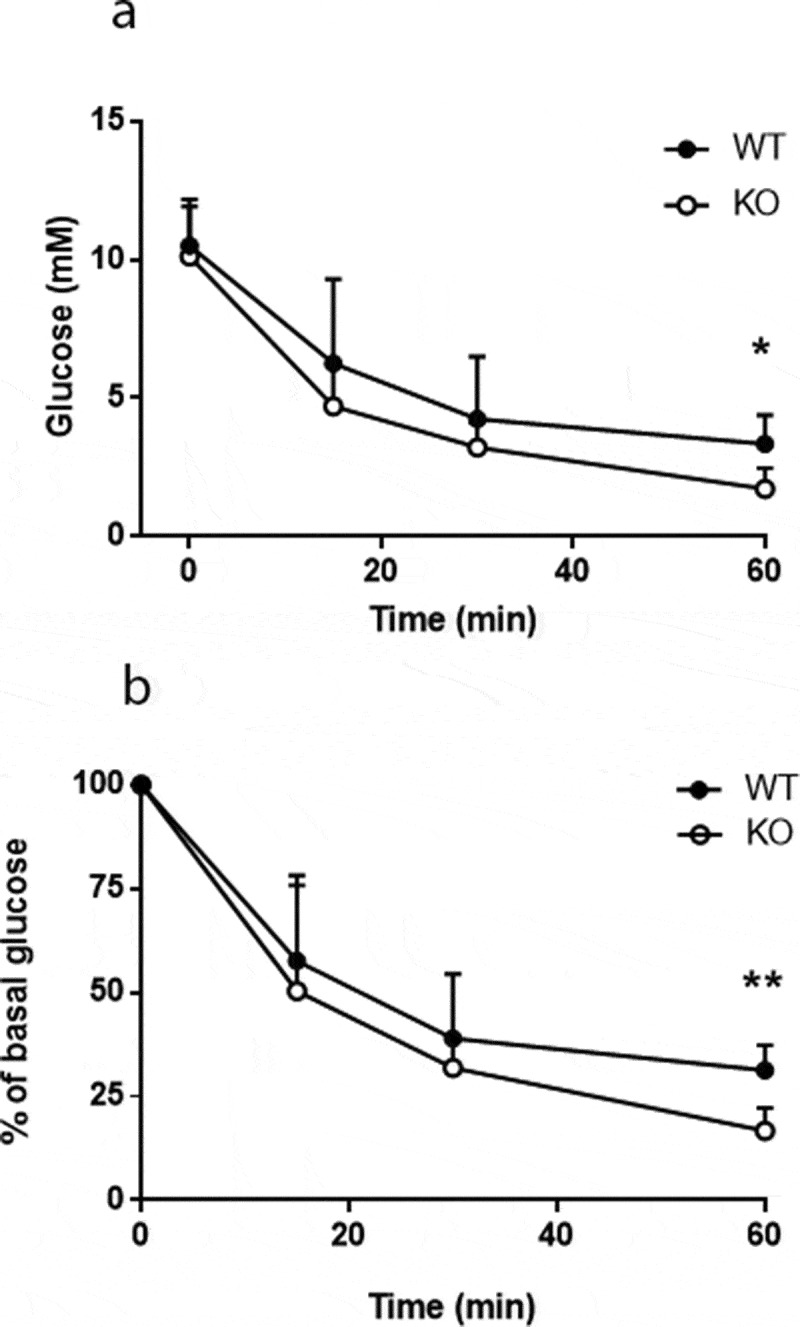
Enhanced insulin sensitivity in Cyp8b1^−/-^ (KO) mice. Blood glucose levels in response to an intraperitoneal insulin tolerance test in HFD-fed Cyp8b1^+/+^ (WT) and Cyp8b1^−/-^ (KO) mice shown as absolute (a) and relative (b) values. Data is shown as mean ± SD, n = 5. ** = p < 0.01

An intraperitoneal injection of insulin showed that glucose was eliminated more rapidly in *Cyp8b1^−/-^* compared to wildtype *Cyp8b1^+/+^* (Figure 6), indicating that *Cyp8b1^−/-^*mice exhibit enhanced insulin sensitivity.

### Mitochondrial density of brown adipocytes is increased in Cyp8b1^−/-^ mice

**Figure 7.**
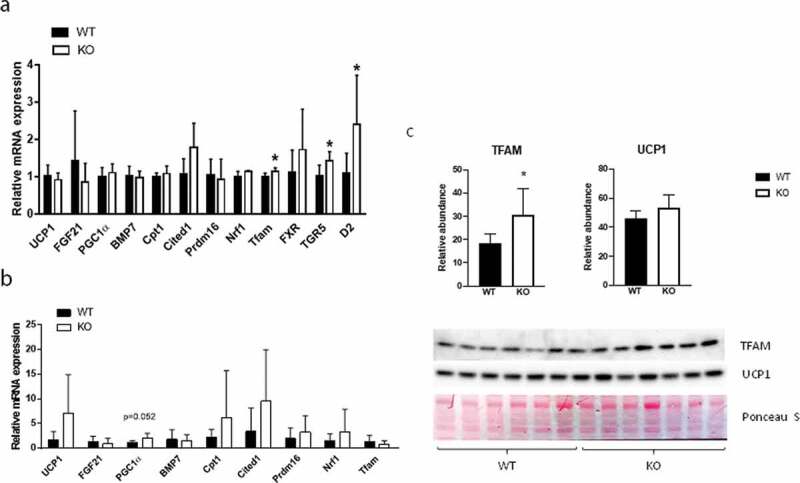
Gene and protein expression in BAT and WAT of Cyp8b1^+/+^ (WT) and Cyp8b1^−/-^ (KO) mice. mRNA levels were analysed with real-time quantitative PCR in (a) interscapular BAT and (b) subcutaneous WAT of HFD-fed Cyp8b1^+/+^ (WT) and Cyp8b1^−/-^ (KO) mice. (c) Protein levels of TFAM and UCP1 were measured by Western blot analysis. The intensity of the respective band was quantified and 567normalized to the intensity of the Ponceau S staining. Data is shown as mean ± SD, n = 5–7. * = p < 0.05

To gain insight into possible mechanisms underlying the observed increased energy expenditure in *Cyp8b1^−/-^* mice, we analysed the expression of genes implicated in BAT thermogenesis and browning of white adipose tissue (WAT), respectively, using qPCR. In interscapular BAT, we found increased expression of TFAM, a transcription factor that promotes mitochondrial biogenesis, and type 2 iodothyronine deiodinase (D2), the enzyme catalysing the conversion of inactive thyroxine (T4) to active 3,5,3ʹ-tri-iodothyronine (T3), in *Cyp8b1^−/-^* mice compared to wildtype mice (Figure 7(a)).

**Figure 8.**
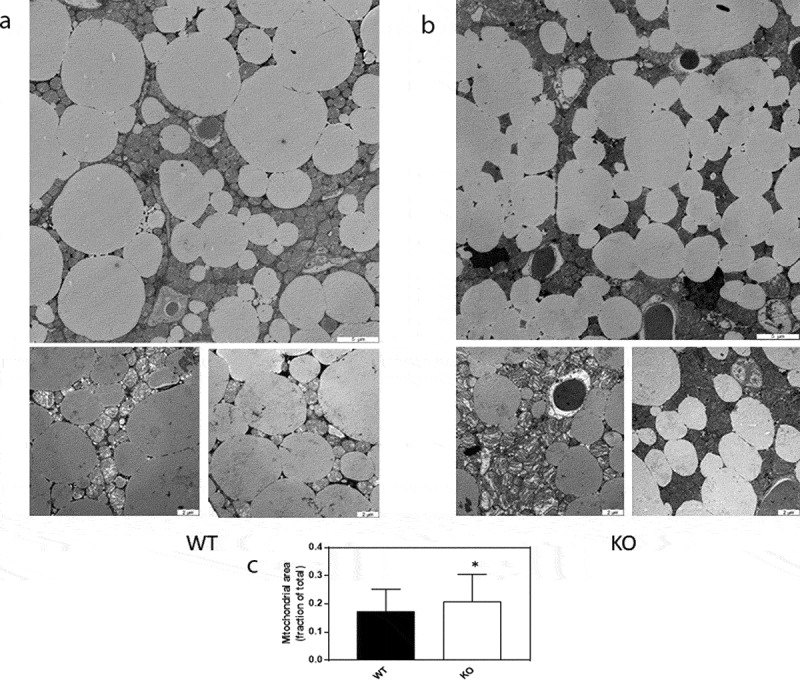
Increased mitochondrial density in BAT of Cyp8b1^−/-^ (KO) mice. 570 Representative transmission electron microscope images of BAT of (a) Cyp8b1^+/+^ (WT) and (b) Cyp8b1^−/-^ (KO) mice. The top image for each genotype shows a larger area. These images have been stiched from four smaller images. (c) Quantification of mitochondrial area relative to total area. Data is shown as mean ± SD and is based on 50 images per genotype (10 images from 5 different animals). * = p < 0.05

In addition, TGR5 was upregulated in *Cyp8b1^−/-^* mice (Figure 7(a)). In subcutaneous WAT, there were no differences in the expression of a selection of genes implicated in browning, although PGC-1α was close to be significantly upregulated in *Cyp8b1^−/^*mice (Figure 7(b)). Western blot analysis of BAT showed higher levels of TFAM protein and a trend towards higher levels of UCP1 protein in *Cyp8b1^−/-^* mice compared to wildtype mice (Figure 7(c)). Next, we used transmission electron microscopy to analyse BAT with regard to mitochondrial density and morphology. We found a 21% increase in mitochondrial density in *Cyp8b1^−/-^* mice compared to wildtype mice (Figure 8). Mitochondrial morphology, analysed blinded and independently by four investigators, revealed no differences between the two groups of mice.

### White adipocytes of Cyp8b1^−/-^ mice exhibit increased lipolytic and anti-lipolytic responsiveness

**Figure 9.**
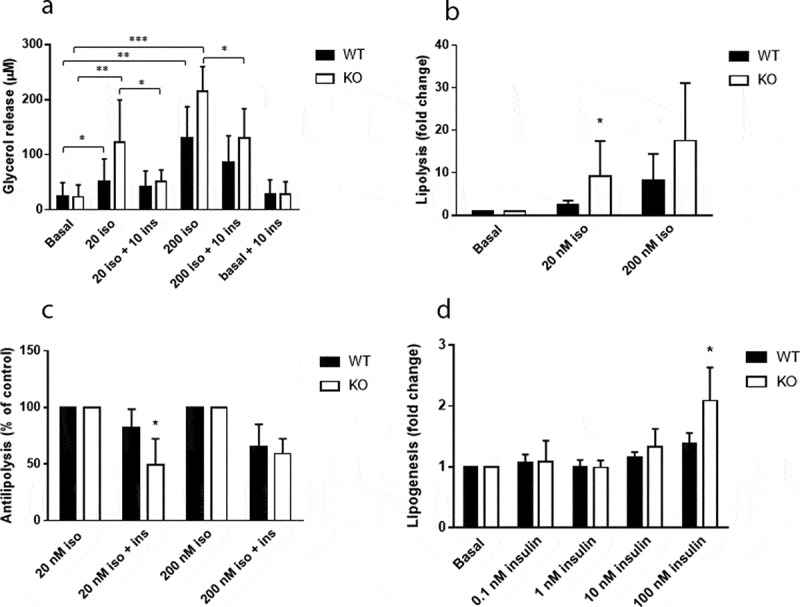
Increased responsiveness to catecholamines and insulin in white adipocytes of Cyp8b1^−/-^ (KO) mice. Adipocytes were isolated from epididymal fat pads of Cyp8b1^+/+^ (WT) and Cyp8b1^−/-^ (KO) mice and used for measurement of lipolysis and lipogenesis. (a) Cells were stimulated with isoproterenol and/or insulin at the indicated concentrations (given in nM) for 30 min. Lipolysis was measured as glycerol concentration of the medium. (b) Isoproterenol-stimulated lipolysis shown as fold change compared to basal lipolysis for respective group. (c) Antilipolytic effect of insulin shown as percent lipolysis in the presence of both isoproterenol and insulin compared to isoproterenol alone. (d) *De novo* lipogenesis was measured during 45 min in the absence or presence of insulin at the indicated concentrations. Insulin stimulated lipogenesis is shown as fold change compared to basal lipogenesis for respective group. Data is shown as mean ± SD, n = 7 (lipolysis) and 8 (lipogenesis), respectively, for both groups. * = p < 0.05

To investigate insulin sensitivity at the level of adipose tissue, we performed measurements of lipolysis and lipogenesis on epididymal adipocytes. There were no differences in basal lipolysis between the two groups, whereas isoproterenol-induced lipolysis was increased in *Cyp8b1^−/-^* mice (Figure 9(a, b)). The ability of insulin to antagonize isoproterenol-induced lipolysis was increased in *Cyp8b1^−/-^* mice, although only significant when lipolysis was induced with the lower isoproterenol concentration (Figure 9(a, c)). Insulin-stimulated lipogenesis was increased in *Cyp8b1^−/^*mice, although statistically significant only at the highest insulin concentration (Figure 9(d)).

## Discussion

Here we show that *Cyp8b1^−/-^* mice are protected against diet-induced obesity, have increased energy expenditure and increased faecal energy output. Furthermore, gene and protein expression analyses of BAT indicated increased mitochondrial biogenesis in *Cyp8b1^−/-^* mice and increased mitochondrial density of brown adipocytes was indeed found in analyses by transmission electron microscopy. Meal tolerance tests and insulin tolerance tests indicated that insulin sensitivity is enhanced in *Cyp8b1^−/^*mice. This was accompanied by enhanced responsiveness of white adipocytes to both catecholamines and insulin. The protection against diet-induced obesity of *Cyp8b1^−/-^* mice is in agreement with two previous studies [6,7]. In addition, the increase in faecal energy output is in agreement with these two studies, although one of them, analysing faecal lipids rather than total faecal energy, showed a significant increase for cholesterol only [6]. Using indirect gas-calorimetry, we found that energy expenditure was increased in *Cyp8b1^−/-^*mice, compared to *Cyp8b1^+/+^*mice. This is in contrast to a previous study showing no change in energy expenditure of *Cyp8b1^−/-^* mice compared to controls [7]. The reason for the discrepancy is not known, but one possibility is the difference in dietary regimen. Our mice were fed a lard-based high-fat diet (60 E%) for 10–14 weeks, whereas Bertaggia *et al.* employed a Western-type diet with 42 E% from fat and 0.2% cholesterol for 4 weeks [7]. A difference in temperature at which the calorimetry experiments were performed is also possible, but the temperature at which the measurements were performed in the study by Bertaggia *et al*. was not stated [7]. We found no differences in food intake between *Cyp8b1^−/-^* mice and control mice, which is in agreement with previous studies [6,7]. Moreover, the lack of difference in RER between the two groups of mice, found both in the present study and the study by Bertaggia *et al.* [7] indicates that substrate utilization is not changed in *Cyp8b1^−/-^*mice.

As a first step to identify mechanisms underlying the increased energy expenditure of *Cyp8b1^−/-^* mice, we focused on the adipose tissues and performed gene expression analyses of WAT and BAT, respectively. Several key players in browning, including PGC-1α, tended to be upregulated in subcutaneous WAT. Although not significant at this point, these results warrant further analyses in a larger material and with complementary techniques. In BAT we found higher expression of TFAM, TGR5 and D2. The upregulation of TFAM is supported by Western blot analyses, where TFAM protein levels were shown to be higher in *Cyp8b1^−/-^* mice. Also UCP1 protein levels tended to be higher in *Cyp8b1^−/-^* mice compared to wildtype mice. The upregulation of TFAM was accompanied by a 21% increase in mitochondrial density, as assessed by transmission electron microscopy. Bile acid-induced increase in the expression of TFAM and higher mitochondrial density is in agreement with a recent report, which demonstrated that activation of TGR5 with taurolithocholic acid induced increased TFAM expression and mitochondrial mass in human aortic endothelial cells [12].

TGR5 and D2 are highly co-expressed in BAT and treatment of HFD-fed mice with cholic acid has been shown to increase energy expenditure via a mechanism involving activation of TGR5 and induction of D2 activity in BAT [13]. The increase in energy expenditure prevented diet-induced obesity and insulin resistance. Results from *in vitro* experiments in the same study showed that the induction of D2 activity was much more prominent in cells obtained from mice fed a high-fat diet (60 E%) than cells from mice fed a normal chow diet [13]. In a more recent study, the increase in energy expenditure induced by administration of cholic acid was shown to be dependent on augmented expression of UCP1 in BAT. In line with induction of energy expenditure by bile acids, lowering of the bile acid pool via treatment with the FXR-agonist GW4064 was shown to result in decreased energy expenditure, weight gain and insulin resistance [14]. Increased energy expenditure following treatment with bile acids has been demonstrated also in humans. Broeders *et al.* showed that treatment of twelve healthy individuals with CDCA for 2 days resulted in higher BAT activity and whole-body energy expenditure [15]. Studies of human brown adipocytes *in vitro* furthermore demonstrated that treatment with 30 µM CDCA enhanced expression of D2 and UCP1, whereas concentrations of 3 µM did not. The plasma concentration of CDCA in *Cyp8b1^−/-^* mice has been reported to be around 5 µM [4] and may thus not be high enough to induce a robust rise in the expression of D2 and UCP1 in this model. Besides the increase in CDCA, there are other changes in the composition of the bile acid pool of *Cyp8b1^−/-^* mice. Cholic acid and other 12-hydroxylated bile acids are obviously lacking, whereas muricholic acids, lithocholic acid and taurocholic acid are increased [3,4,7]. Litocholic acid and taurolithocholic acid are the major ligands for TGR5 [16] and muricholic acids have been shown to act as FXR antagonists [17]. More studies are needed in order to elucidate the link between the phenotype of *Cyp8b1^−/-^* mice and the changes in the size and composition of the bile acid pool. However, it is tempting to speculate that there is a relative increase in TGR5 signalling and a relative decrease in FXR signalling in *Cyp8b1^−/-^* mice compared to wildtype mice. Several different metabolic tests were performed to assess glucose homoeostasis in *Cyp8b1^−/-^* mice. First, a mixed meal tolerance test was used. This is a more physiological test than the oral glucose tolerance test, since it takes into account other insulin secretagogues than glucose. We found that whereas glucose disposal was not different between the groups, the insulin response was lower in *Cyp8b1^−/-^* mice than in *Cyp8b1^+/+^*mice. This could reflect improved insulin action in *Cyp8b1^−/-^* mice.

Enhanced insulin sensitivity in *Cyp8b1^−/-^* mice was indeed found when assessed by insulin tolerance tests, in agreement with previous studies [4,6]. As a third test, we performed an IVGTT, which circumvents several processes that influence glucose disposal during meal tolerance tests and oral glucose tolerance tests, such as gastric emptying and the incretin effect. In the IVGTT, we found no differences in neither glucose disposal nor insulin response between *Cyp8b1^−/-^* mice and *Cyp8b1^+/+^*mice. Finally, insulin secretion, including exacerbation to the incretin GLP-1, was not different between *Cyp8b1^−/-^* mice and *Cyp8b1^+/+^*mice, as assessed by static incubations of isolated islets. Oral glucose tolerance tests have been used in previous studies to demonstrate improved glucose tolerance in *Cyp8b1^−/-^* mice [4,6,7]. These reports show an improvement in glucose disposal in *Cyp8b1^−/-^* mice compared to *Cyp8b1^+/+^*mice. Insulin levels, however, were not reported, making it difficult to evaluate these results in the light of ours. Taken together, the metabolic tests indicate that insulin sensitivity is enhanced in *Cyp8b1^−/-^* mice, whereas there seem to be no differences between the groups with regard to glucose-stimulated insulin secretion. Future studies will have to address whether effects on gastric emptying and/or plasma levels of GLP-1 influence glucose homoeostasis in *Cyp8b1^−/-^* mice. Interestingly, upregulation of TGR5 and nNOS in gastric myenteric plexa, resulting in delayed gastric emptying, has been demonstrated in HFD-fed rats [18]. Moreover, increased levels of GLP-1 in plasma has been described in *Cyp8b1^−/-^* mice [4,6], and GLP-1 may influence glucose homoeostasis in many different ways, including retardation of gastric emptying [19].

In order to start identifying mechanisms underlying the enhanced insulin sensitivity, we focused on adipose tissue. Experiments performed on isolated white adipocytes demonstrated increased responsiveness to both lipolytic (isoproterenol) and antilipolytic (insulin) stimuli, whereas basal lipolysis was unaffected. The improved responsiveness to insulin may in fact have been underestimated because responsiveness is increased also towards catecholamines. In agreement with the effects on lipolysis, insulin-stimulated lipogenesis was enhanced in *Cyp8b1^−/-^* mice, although significant only at the higher insulin concentrations. Detailed studies of receptor and post-receptor events will have to be performed to further define the mechanisms that underlie the effects on lipolytic responsiveness in *Cyp8b1^−/-^* mice and, furthermore, if the increased levels of GLP-1 [4] contribute to these effects.

Besides increased energy expenditure, decreased intestinal lipid absorption, as reflected in increased faecal energy output, most likely contributes to the protection against diet-induced obesity in *Cyp8b1^−/-^* mice. Whether effects on intestinal lipid absorption also contributes to the improvements in glucose homoeostasis in *Cyp8b1^−/^*mice is still unclear. It has been suggested that the higher fatty acid content of ileum found in *Cyp8b1^−/-^* mice, accounts for increased secretion of GLP-1 from L-cells [4]. An incidental finding made during the course of this study, was the high lethality at birth of pups born from homozygous breeding. We speculate that this may be due to the recently demonstrated role of bile acids as chaperones in the development of haematopoietic stem cells [20]. The work by Sigurdsson *et al.* demonstrates the coordinated regulation of haematopoiesis in the foetal liver by foetal and maternal bile acids. Furthermore, they demonstrate that a derivative of cholic acid, taurocholic acid, appears to be main player in this regulation [20]. Thus, complete absence of cholic acid, as in pups born from homozygous breeding, may not protect haematopoietic stem cells from unfolded protein stress in the foetal liver, whereas the very low amounts in pups born from heterozygous breeding do. In conclusion, we show that *Cyp8b1^−/-^* mice have increased energy expenditure. We also demonstrate higher mitochondrial density of brown adipocytes, as one possible mechanism underlying the higher energy expenditure. Furthermore, we demonstrate that enhanced insulin sensitivity in *Cyp8b1^−/-^* mice is accompanied by increased responsiveness of white adipocytes to both catecholamines and insulin. Changes in the size and/or composition of the bile acid pool could be a strategy to prevent and treat obesity and insulin resistance.

## References

[1] Molinaro A, Wahlstrom A, Marschall HU. Role of bile acids in metabolic control. Trends Endocrinol Metab. 2018;29(1):31–41. 29195686 10.1016/j.tem.2017.11.002

[2] Schaap FG, Trauner M, Jansen PL. Bile acid receptors as targets for drug development. Nat Rev Gastroenterol Hepatol. 2014;11(1):55–67. 23982684 10.1038/nrgastro.2013.151

[3] Li-Hawkins J, Gafvels M, Olin M, et al. Cholic acid mediates negative feedback regulation of bile acid synthesis in mice. J Clin Invest. 2002;110(8):1191–1200. 12393855 10.1172/JCI16309 PMC150802

[4] Kaur A, Patankar JV, de Haan W, et al. Loss of Cyp8b1 improves glucose homeostasis by increasing GLP-1. Diabetes. 2015;64(4):1168–1179. 25338812 10.2337/db14-0716

[5] Rudling M. Understanding mouse bile acid formation: is it time to unwind why mice and rats make unique bile acids? J Lipid Res. 2016;57(12):20978. 10.1194/jlr.C072876 PMC5321220 27777318

[6] Bonde Y, Eggertsen G, Rudling M. Mice abundant in muricholic bile acids show resistance to dietary induced steatosis, weight gain, and to impaired glucose metabolism. PLoS One. 2016;11(1):e0147772. 26824238 10.1371/journal.pone.0147772 PMC4732983

[7] Bertaggia E, Jensen KK, Castro-Perez J, et al. Cyp8b1 ablation prevents Western diet-induced weight gain and hepatic steatosis because of impaired fat absorption. Am J Physiol Endocrinol Metab. 2017;313(2):E121–E33. 28377401 10.1152/ajpendo.00409.2016 PMC5582885

[8] Brommage R. Validation and calibration of DEXA body composition in mice. Am J Physiol Endocrinol Metab. 2003;285(3):E454–9. 12759224 10.1152/ajpendo.00470.2002

[9] Ahren B, Simonsson E, Scheurink AJ, et al. Dissociated insulinotropic sensitivity to glucose and carbachol in high-fat diet-induced insulin resistance in C57BL/6J mice. Metabolism. 1997;46(1):97–106. 9005977 10.1016/s0026-0495(97)90175-x

[10] Clark AM, Sousa KM, Jennings C, et al. Continuous flow enzyme assay on a microfluidic chip for monitoring glycerol secretion from cultured adipocytes. Anal Chem. 2009;81(6):2350–2356. 19231843 10.1021/ac8026965 PMC2659456

[11] Moody AJ, Stan MA, Stan M, et al. A simple free fat cell bioassay for insulin. Horm Metab Res. 1974;6(1):12–16. 4819286 10.1055/s-0028-1093895

[12] Zhao LJ, Zhang SF. Activation of TGR5 promotes mitochondrial biogenesis in human aortic endothelial cells. Biochem Biophys Res Commun. 2018;500(4):952–957. 29709472 10.1016/j.bbrc.2018.04.210

[13] Watanabe M, Houten SM, Mataki C, et al. Bile acids induce energy expenditure by promoting intracellular thyroid hormone activation. Nature. 2006;439(7075):484–489. 16400329 10.1038/nature04330

[14] Watanabe M, Horai Y, Houten SM, et al. Lowering bile acid pool size with a synthetic farnesoid X receptor (FXR) agonist induces obesity and diabetes through reduced energy expenditure. J Biol Chem. 2011;286(30):26913–26920. 21632533 10.1074/jbc.M111.248203 PMC3143650

[15] Broeders EP, Nascimento EB, Havekes B, et al. The Bile Acid Chenodeoxycholic Acid Increases Human Brown Adipose Tissue Activity. Cell Metab. 2015;22(3):418–426. 26235421 10.1016/j.cmet.2015.07.002

[16] Prawitt J, Caron S, Staels B. Bile acid metabolism and the pathogenesis of type 2 diabetes. Curr Diab Rep. 2011;11(3):160–166. 21431855 10.1007/s11892-011-0187-x PMC3338411

[17] Hu X, Bonde Y, Eggertsen G, et al. Muricholic bile acids are potent regulators of bile acid synthesis via a positive feedback mechanism. J Intern Med. 2014;275(1):27–38. 24118394 10.1111/joim.12140

[18] Zhou H, Zhou S, Gao J, et al. Upregulation of bile acid receptor TGR5 and nNOS in gastric myenteric plexus is responsible for delayed gastric emptying after chronic high-fat feeding in rats. Am J Physiol Gastrointest Liver Physiol. 2015;308(10):G863–73. 25540233 10.1152/ajpgi.00380.2014 PMC4437020

[19] Nauck MA, Meier JJ. Incretin hormones: their role in health and disease. Diabetes Obes Metab. 2018;20(Suppl 1):5–21. 29364588 10.1111/dom.13129

[20] Sigurdsson V, Takei H, Soboleva S, et al. Bile acids protect expanding hematopoietic stem cells from unfolded protein stress in fetal liver. Cell Stem Cell. 2016;18(4):522–532. 26831518 10.1016/j.stem.2016.01.002

